# Detection of PD-L1 expression levels in malignant pleural mesothelioma with a targeted MRI nanoprobe *in vivo*

**DOI:** 10.3389/fchem.2024.1508912

**Published:** 2024-12-02

**Authors:** Zhenghua Zhang, Yang Tian, Wenjun Gao, Yubin Hu, Liangping Luo, Lichang Lei, Shasha Shen, Dan Han

**Affiliations:** ^1^ Medical Imaging Department, The First Affiliated Hospital of Kunming Medical University, Kunming, China; ^2^ Department of Radiology, First Affiliated Hospital of Jinan University, Guangzhou, Guangdong, China

**Keywords:** malignant pleural mesothelioma (MPM), immunotherapy, programmed death ligand 1 (PD-L1), magnetic resonance imaging (MRI), nanoprobe

## Abstract

**Objectives:**

Immune checkpoint inhibitors (ICIs) have demonstrated potential in inhibiting the growth of malignant pleural mesothelioma (MPM), and their efficacy is associated with the expression of programmed death-ligand 1(PD-L1). This study evaluated a PD-L1-targeted nanoprobe for detecting PD-L1 expression in a nude mouse model of malignant pleural mesothelioma (MPM).

**Methods:**

A PD-L1-binding peptide (WL-12) was conjugated with superparamagnetic iron oxide nanoparticles (SPIONs) to create the nanoprobe WL-12@Fe₃O₄. The nanoprobe’s stability, biotoxicity, targeting ability, and *in vivo* magnetic resonance (MR) imaging effects were assessed and compared to non-targeted Fe₃O₄ nanoparticles. ΔT2 values and PD-L1 expression were measured in H226 and MSTO-211H tumor tissues over 4 weeks to analyze correlations.

**Results:**

The WL-12@Fe₃O₄ nanoprobe demonstrated uniform distribution and a spherical shape, with a larger size (43.82 nm) and lower surface potential (−9.34 ± 0.54 mV) compared to Fe₃O₄ (32.67 nm, −20.20 ± 0.88 mV, *P* < 0.05). The XPS and FT-IR analysis results indicate the successful coupling of WL-12 with Fe_3_O_4._ It was well dispersed in serum and saline and showed no cytotoxicity or organ damage *in vivo*. The probe selectively accumulated in PD-L1-expressing MPM cells, especially MSTO-211H, and exhibited significantly higher uptake in high PD-L1-expressing H460 cells (930.22 ± 11.75 ng/mL) compared to low PD-L1-expressing A549 cells (254.89 ± 17.33 ng/mL, *P* < 0.05). Tumor iron levels in the WL-12@Fe₃O₄ group were significantly elevated (141.02 ± 17.33 μg/g) compared to controls (36.43 ± 3.56 μg/g, *P* < 0.05), with no significant differences in other organs (*P* > 0.05). The T2 values of H226 and MSTO-211H tumors decreased after probe injection, with ΔT2 values significantly higher in the targeted group than the nontargeted group (*P* < 0.05). ΔT2 values increased over 4 weeks, correlating strongly with PD-L1 expression (*P* < 0.05).

**Conclusion:**

The PD-L1-targeted nanoprobe with MRI is a promising tool for noninvasive, real-time assessment of PD-L1 expression in MPM.

## 1 Introduction

Malignant pleural mesothelioma (MPM) is a rare but highly aggressive tumor originating from pleural mesothelial cells and is closely associated with asbestos exposure. Owing to its insidious onset, most patients are diagnosed at an advanced stage, resulting in limited treatment options. The average survival time for MPM patients ranges from 6.8 to 17.2 months, with a median overall survival of approximately 1 year and a 5-year survival rate of approximately 10%, whereas cases of complete cure are exceedingly rare (Wang et al., 2023; van Kooten et al., 2022). A phase III clinical trial indicated that immunotherapy with nivolumab and ipilimumab significantly improved overall survival in MPM patients compared with chemotherapy (including platinum-based agents and/or pemetrexed), with a 2-year overall survival rate of 41% versus 27% (Baas et al., 2021).

In various malignancies, immune checkpoint inhibitors (ICIs) have been proven to have significant antitumour effects, and their efficacy is associated with the expression of programmed death-ligand 1 (PD-L1) (Hegde and Chen, 2020). However, PD-L1 expression in MPM often displays spatial and temporal heterogeneity, including intratumoral heterogeneity and changes during the treatment course (Terra et al., 2017). Compared with immunohistochemistry (IHC), there is an urgent need to develop a technique that allows real-time, quantitative, and noninvasive assessment of PD-L1 expression in tumors.

A study utilizing the 99mTc-MY1523 SPECT/CT technique has successfully achieved real-time, quantitative monitoring of PD-L1 expression within tumors, providing valuable guidance for PD-L1 blockade therapy and significantly improving its efficacy (Gao et al., 2020). However, imaging with radiolabeled antibodies is associated with considerable background signal, requiring imaging several days after the injection of monoclonal antibodies to achieve optimal contrast, and involves radiation exposure. In recent years, MRI has made significant advancements, offering excellent temporal and spatial resolution, and has shown great potential in the imaging of immune biomarkers (Lee et al., 2021).

Our research team previously explored the potential of multiparametric MRI in evaluating the pathological types of MPM and successfully synthesized a mesothelin-targeted nanoprobe for the diagnosis and differential diagnosis of MPM (Zhang Z. et al., 2023; Huang et al., 2024). Building on these findings, this study aimed to synthesize an MRI nanoprobe targeting PD-L1 and evaluate its feasibility for detecting PD-L1 expression in a nude mouse MPM xenograft model.

## 2 Materials and methods

### 2.1 Reagents

Iron (III) acetylacetonate, dibenzyl ether, oleylamine, anhydrous ethanol, and the WL-12 peptide were obtained from Shanghai Carboxphyll Bio-Tech Co., Ltd. 1-Ethyl-3-(3-dimethylaminopropyl) carbodiimide (EDC) and N-hydroxysuccinimide (NHS) were supplied by Aladdin Bio-Tech Co., Ltd. All other chemicals were received in good amounts and were of reagent quality.

### 2.2 Synthesis of superparamagnetic iron oxide nanoparticles (SPIONs)

In the synthesis process, iron (III) acetylacetonate (2 g), dibenzyl ether (20 mL), and oleylamine (20 mL) were mixed in a dry three-neck round-bottom flask. While stirring the mixture, nitrogen gas was slowly introduced to create an inert atmosphere. The mixture was then placed on a heated magnetic stirrer and stirred to ensure homogeneity. The temperature was gradually increased to 100°C and maintained for 1 h to ensure complete dissolution of the components.

Next, the temperature was gradually increased to 295°C at a rate of 5°C every 10 min. Once the temperature reached 295°C, the reaction was allowed to proceed for 30 min to ensure completion. After the reaction was complete, the heat was turned off, and the mixture was allowed to cool naturally to room temperature.

The Fe₃O₄ nanoparticles were subsequently separated via an external magnetic field, and the product was subsequently washed several times with anhydrous ethanol until the supernatant became clear. The washed product was then placed in a vacuum drying oven and dried at 60°C for 12 h, yielding the final SPION.

### 2.3 Synthesis of WL-12@Fe_3_O_4_

First, 1-ethyl-3-(3-dimethylaminopropyl)carbodiimide (EDC) and N-hydroxysuccinimide (NHS) were dissolved in phosphate-buffered saline (PBS) and mixed at a 1:1 M ratio. The mixture was then subjected to continuous stirring at room temperature for 1 h to facilitate the reaction. Next, an equimolar amount of the Fe_3_O_4_ nanoparticle dispersion was added to the reaction mixture, and the resulting solution was incubated overnight at 4°C.

After the reaction, the mixture was centrifuged at high speed to remove the supernatant, and the precipitate was washed three times with distilled water, with centrifugation performed after each wash. The supernatant was then discarded, and the precipitate was resuspended in PBS. Subsequently, WL-12 was dissolved in PBS at a molar ratio of 2:1 (WL-12:Fe_3_O_4_ nanoparticles) and reacted with the Fe_3_O_4_ nanoparticle suspension at 4°C overnight.

Finally, the covalently bound product was subjected to centrifugation and filtration, followed by three additional washes with distilled water to remove any residual reactants. The resulting precipitate was freeze-dried to obtain WL-12@Fe_3_O_4_.

### 2.4 Characterization of the materials

The morphology and distribution of the nanoparticles were examined by scanning electron microscopy (SEM). Fe₃O₄ and WL-12@Fe₃O₄ nanoparticles (1 mg each) were separately dissolved in 2 mL of deionized water, followed by ultrasonic treatment at 40% power for three cycles, each lasting 5 min. A 50 µL aliquot of the resulting suspension was then diluted with 2 mL of deionized water for further analysis. Dynamic light scattering (DLS) was employed to measure the particle size distribution and zeta potential of the nanoparticles.

The WL-12@Fe_3_O_4_ nanoparticles were diluted with saline and 100% FBS solutions to different concentrations (0, 20, 40, 60, 80, and 160 μg/mL) and placed into EP tubes with a total volume of 1 mL per tube. The Zeta potential of the WL-12@Fe_3_O_4_ nanoparticles was measured at 0, 12, and 24 h at room temperature to assess stability.

#### 2.4.1 X-ray photoelectron spectroscopy (XPS)

A suitable amount of the sample was pressed into a pellet and affixed to the sample holder. The sample was then placed into the sample chamber of the Thermo Scientific K-Alpha XPS instrument. Once the pressure in the sample chamber was reduced to less than 2.0 × 10^−7^ mbar, the sample was transferred to the analysis chamber. The analysis was conducted with a spot size of 400 μm, operating at a voltage of 12 kV and a filament current of 6 mA. Wide-scan spectra were acquired with a pass energy of 150 eV and a step size of 1 eV. Narrow-scan spectra were obtained with a pass energy of 50 eV and a step size of 0.1 eV.

#### 2.4.2 Fourier transform infrared spectroscopy (FT-IR)

The instrument used was a Thermo Scientific Nicolet iS20. Under a dry environment, a visually discernible amount of the sample and an appropriate quantity of dried potassium bromide powder were placed in a mortar and ground thoroughly multiple times. The mixture was then pressed into transparent pellets using a pellet press. During the FT-IR analysis, the background spectrum was collected first, followed by the sample’s infrared spectrum. The resolution was set to 4 cm^-1^, with 32 scans, and the spectral range was from 400 to 4000 cm^−1^.

### 2.5 Cell culture

The cell lines used in this study included the human epithelial-type MPM cell line H226, the human biphasic MPM cell line MSTO-211H, and the human lung cancer cell lines A549 and H460, all of which were obtained from the Cell Bank of the Typical Culture Preservation Committee of the Chinese Academy of Sciences. All the cells were cultured in RPMI 1640 medium supplemented with 10% FBS, 10,000 U/mL penicillin, and 10 mg/mL streptomycin under standard conditions (37°C and 5% CO_2_).

### 2.6 Establishment of a nude mouse MPM xenograft model

Female BALB/c nude mice (four to six weeks old, SCXK (Dian) K2020-004) were purchased from the Laboratory Animal Department of Kunming Medical University. The mice were housed under specific pathogen-free (SPF) conditions with *ad libitum* access to sterile food and water and maintained on a 12-h light/dark cycle, with the temperature and humidity controlled at 24°C–26°C and 30%–50%, respectively. The animal experiments in this study were approved by the Animal Experiment Ethics Review Committee of Kunming Medical University (Approval No. KMMU20220931).

H226 and MSTO-211H cells in the logarithmic growth phase were harvested via trypsinization with 0.25% trypsin. After centrifugation, the cells were resuspended in saline, and the cell suspension concentration was adjusted to 12 × 10^6^ cells/50 µL. The cell suspension was mixed with Matrigel (50 µL) and subcutaneously injected into the right axilla of the nude mice. The body weights and tumor volumes of the tumor-bearing mice were recorded daily, and tumor growth was monitored for an additional 4 weeks once the tumors reached a maximum diameter of 1 cm.

### 2.7 Toxicity assessment of WL-12@Fe_3_O_4_

#### 2.7.1 *In vitro* cytotoxicity experiment

H226 and MSTO-211H cells were seeded at a density of 5,000 cells per well in 96-well plates and incubated for 48 h. Once the cell confluency reached approximately 80%, 10 µL of nanoparticle solution (dissolved in culture medium) was added to each well at final concentrations of 0, 25, 50, 100, and 200 μg/mL. The cells were then incubated for an additional 24 h. After the incubation period, 10 µL of CCK-8 solution was added to each well and incubated for another 2 h. The optical density (OD) of each sample was measured via a microplate reader at a wavelength of 450 nm. Cell viability was calculated via the following formula.

$$\begin{array}{cc} Cell & viability \end{array}=\frac{OD_{sample}-OD_{blank}}{OD_{control}-OD_{blank}}\times 100\%$$

#### 2.7.2 *In vivo* cytotoxicity experiment

One hundred microliters of nanoparticle solution (dissolved in saline at a concentration of 1 mg/mL) was administered to the nude mice via tail vein injection. The mice were sacrificed by cervical dislocation at 1, 2, 3, and 4 weeks postinjection, and major organs, including the brain, heart, lungs, liver, spleen, and kidneys, were harvested. The organs were subjected to hematoxylin and eosin (HE) staining, and the tissue samples were examined under a conventional light microscope.

### 2.8 Targeting study of WL-12@Fe_3_O_4_

H226 and MSTO-211H cells were seeded into 24-well plates at a density of 7 × 10⁴ cells per well and incubated at 37°C with 5% CO₂ for 24 h. After the cells were washed three times with PBS, 30 µL of WL-12@Fe_3_O_4_ nanoparticles or Fe_3_O_4_ nanoparticles (at a concentration of 1 mg/mL) were added to each well, followed by incubation for an additional hour. The deposition of iron particles in both cell types was then observed via Prussian blue staining.

H460 cells (high PD-L1 expression) and A549 cells (low PD-L1 expression) were also seeded into 24-well plates at a density of 7 × 10⁴ cells per well and incubated at 37°C with 5% CO₂ for 24 h. After three PBS washes, 100 µL of WL-12@Fe_3_O_4_ nanoparticles or Fe_3_O_4_ nanoparticles (at a concentration of 1 mg/mL) were added to each well, with the control group receiving regular culture medium. The cells were incubated for an additional 6 h. The concentration of iron in the dialysate was measured via inductively coupled plasma‒mass spectrometry (ICP-MS).

For the *in vivo* targeting study, tumor-bearing mice were injected via the tail vein with 100 µL of WL-12@Fe_3_O_4_ nanoparticles or Fe_3_O_4_ nanoparticles (1 mg/mL, dissolved in saline). After 1 h, the mice were sacrificed by cervical dislocation, and major organs, including the heart, kidneys, liver, lungs, spleen, and tumor tissues, were harvested. The iron content in the tissue lysates was measured via ICP‒MS.

### 2.9 Magnetic resonance imaging (MRI) experiments

After anaesthesia, the tumor-bearing mice were positioned on a 3.0T GE MRI scanner for T1-weighted imaging (T1WI), T2-weighted imaging (T2WI), and T2 mapping. The specific scanning parameters are provided in Table 1. Following the initial scans, 100 µL of WL-12@Fe_3_O_4_ nanoparticles or Fe_3_O_4_ nanoparticles (1 mg/mL concentration) were injected intravenously via the tail vein. The injection concentration was determined on the basis of previous studies (Huang et al., 2024) and related literature review (Shen and Yu, 2023). T2 mapping scans were repeated at 0.5, 1, 1.5, and 2 h postinjection to evaluate the MR imaging effects of nontargeted (Fe_3_O_4_) and targeted nanoparticles (WL-12@Fe_3_O_4_) in H226 and MSTO-211H tumor-bearing mice. The mice were divided into nontargeted and targeted nanoprobe groups, with 5 mice per group and a total of 20 mice. For four consecutive weeks, the ability of targeted MRI to detect PD-L1 expression levels in the two types of tumors was assessed, with 20 mice per group and a total of 40 mice.

**TABLE 1:** MRI scan parameters.

Images	T1WI	T2WI	T2 mapping
Pulse sequence	SE	TSE	TSF
TE (ms)	15	103	9/18/27/36/45/54/63/72
TR (ms)	579	2,288	1,249
SL (mm)	1	1	1
ST (mm)	2	2	2
Matrix	256 × 320	320 × 320	256 × 205
Voxel	0.156	0.125	0.195
FOV(mm^2^)	50 × 40	40 × 40	50 × 40
Flip angle	90°	90°	90°
Number of signal averaged	4	6	1

Note: T1WI,T1-weighted imaging; T2WI, T2-weighted imaging; TE, echo time; TR, time of repetition; SL, slice increment; ST, slice thickness; FOV, field of view.

The raw data from the tumor-bearing mice were processed and analysed via a GE postprocessing workstation (Advantage Windows 4.6, GE Medical Systems, United States). T1WI and T2WI images were used to observe the location, morphology, and size of the tumors. Regions of interest (ROIs) were delineated on the largest tumor slice and two adjacent slices, covering more than 90% of the total tumor area while avoiding necrotic regions. The T2 relaxation time (T2 value) of the tumor tissues was measured via T2 maps. A double-blind method was employed for data measurement on both plain and contrast-enhanced images, with two attending physicians independently conducting measurements. Each physician measured the images three times, and the average value was taken. The T2 difference (ΔT2 value) between the plain and enhanced scans was calculated as follows: ΔT2 = T2 value (unenhanced) - T2 value (enhanced).

### 2.10 Immunohistochemical analysis of PD-L1 expression in tumor tissues

Following MRI scanning, the tumor-bearing mice were euthanized via cervical dislocation, and the tumor tissues were harvested. The tissues were then fixed in 10% neutral formalin solution. Immunohistochemical staining was performed to assess PD-L1 expression levels in the tumor tissues. An Aipathwell AI-based digital pathology image analysis system (provided by Servicebio, China) was used to quantify the target areas under high magnification. The system automatically calculates the proportion of positive cells (percentage of positive cells = number of positive cells/total number of cells × 100%). The average PD-L1 expression levels from the largest tumor section and its two adjacent sections were selected as the study’s metric.

### 2.11 Statistical analyses

Statistical analysis and graphical plotting were performed via SPSS software version 26.00 and R language (version v4.2.1). The data are presented as the means ± standard deviations (SDs). For normally distributed data, an independent sample t-test was used to compare two groups, whereas one-way analysis of variance (ANOVA) was applied for comparisons among multiple groups. For nonnormally distributed data, the Mann‒Whitney U test was used. A *P*-value of <0.05 was considered to indicate statistical significance.

## 3 Results

### 3.1 Characterization of WL-12@Fe_3_O_4_

**FIGURE 1:**
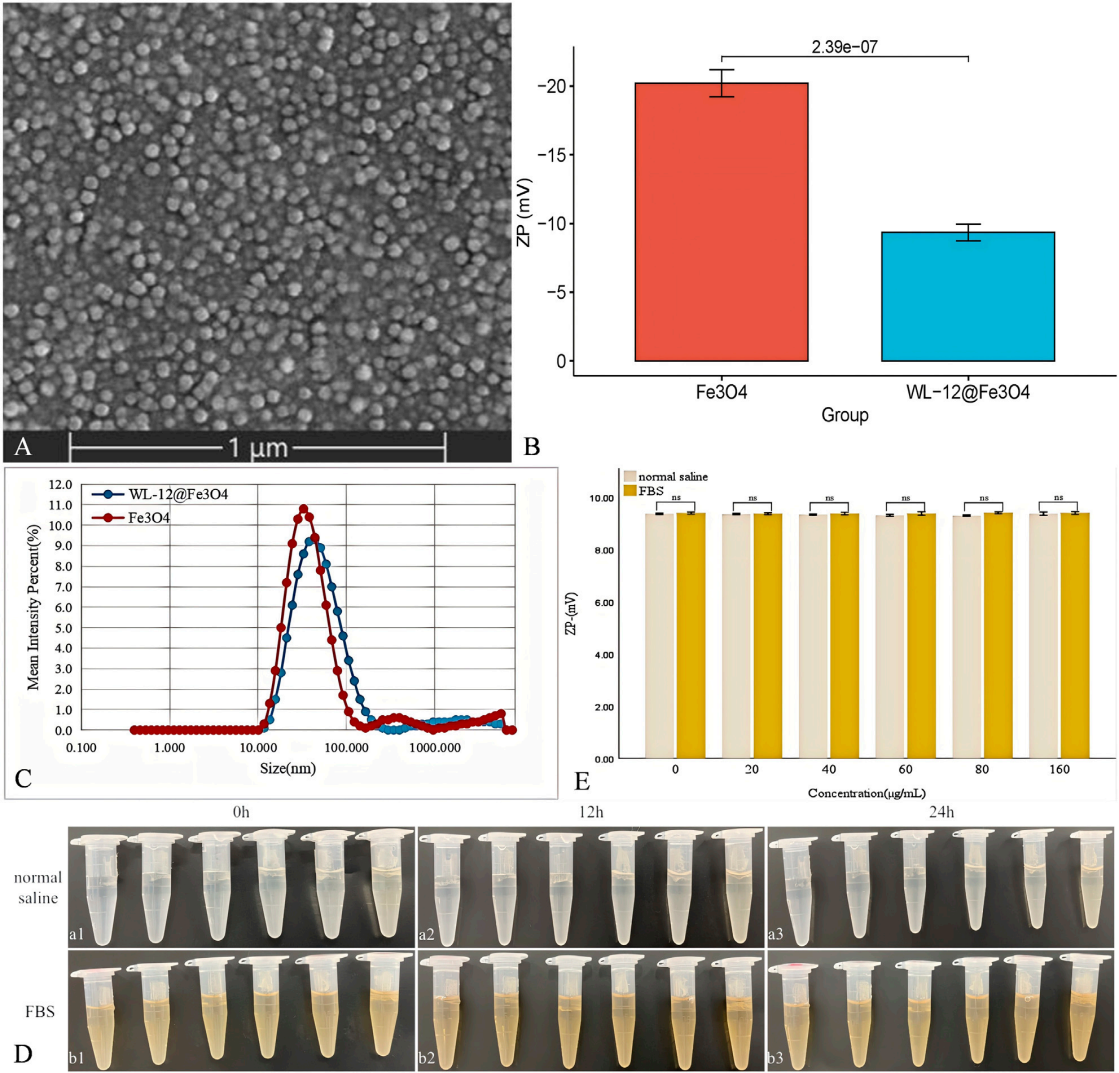
Characterization of the WL-12@Fe_3_O_4_ nanoparticles. **(A)** Scanning electron microscopy (SEM) image of WL-12@Fe_3_O_4_ nanoparticles. **(B)** Comparative bar graph of Zeta potential between WL-12@Fe₃O₄ and Fe₃O₄ nanoparticles. **(C)** Particle size distribution curves of WL-12@Fe_3_O_4_ and Fe_3_O_4_ nanoparticles. **(D)** Comparison of WL-12@Fe₃O₄ nanoparticle solutions at different concentrations. (a: Saline solution. b: FBS solution. From left to right, the concentrations of the nanoparticles are 0, 20, 40, 60, 80, and 160 μg/mL) **(E)**. Comparative bar graph of the average Zeta potential of WL-12@Fe₃O₄ nanoparticles in different solutions over time (ns, *p* > 0.05).

Scanning electron microscopy (SEM) images revealed that the WL-12@Fe_3_O_4_ nanoparticles exhibited a uniform spherical shape and homogeneous distribution (Figure 1A). Compared with that of Fe_3_O_4_, the electron-withdrawing ability of the WL-12@Fe_3_O_4_ nanoparticles decreased, which led to a reduction in the surface potential (−9.34 ± 0.54 mV vs. −20.20 ± 0.88 mV, *P* < 0.05) (Figure 1B). Both WL-12@Fe_3_O_4_ and Fe_3_O_4_ nanoparticles displayed unimodal size distributions. The average particle size of the WL-12@Fe_3_O_4_ nanoparticles (43.82 nm) was larger than that of the Fe_3_O_4_ nanoparticles (32.67 nm) (Figure 1C).

After the WL-12@Fe_3_O_4_ nanoparticles were diluted in physiological saline and 100% FBS, no significant aggregation or precipitation was observed at 0 h, 12 h, or 24 h at room temperature in either medium (Figure 1D). And, the Zeta potential of WL-12@Fe_3_O_4_ nanoparticles in different solutions did not change significantly (*P* > 0.05) (Figure 1E).

**FIGURE 2:**
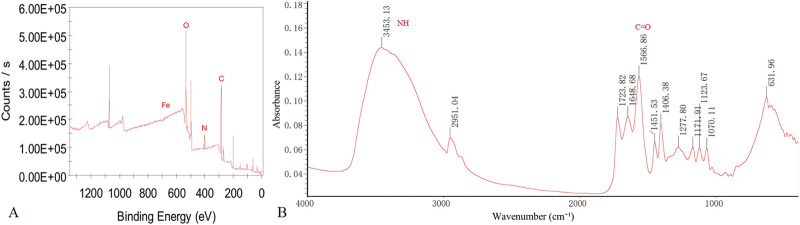
XPS and FT-IR Results of WL-12@Fe_3_O_4_ nanoparticles. **(A)** Full-spectrum XPS analysis. **(B)** FT-IR spectral analysis.

The XPS spectrum reveals the presence of Fe 2p, O 1s, C 1s, and N 1s peaks within the nanoparticles. Notably, the Fe 2p peak predominantly appears around 711.5 eV. When combined with XANES data, this indicates that iron primarily exists in the +3 oxidation state. The FT-IR spectrum demonstrates the occurrence of NH stretching vibration peaks at 3,453.13 cm⁻^1^ and C=O stretching vibrations at 1,566.86 cm⁻^1^ (Figure 2).

### 3.2 Toxicity of WL-12@Fe_3_O_4_ to cells and organs

**FIGURE 3:**
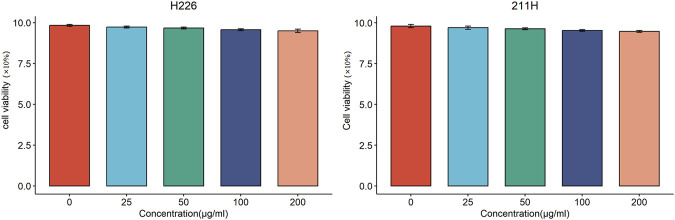
Cytotoxic effects of WL-12@Fe_3_O_4_ nanoparticles on H226 and MSTO-211H cells.

The cytotoxic effects of WL-12@Fe_3_O_4_ nanoparticles on H226 and MSTO-211H cells were evaluated via a CCK-8 assay. Both cell lines were cocultured with various concentrations of nanoparticles (0, 25, 50, 100, or 200 μg/mL) for 24 h, and the cell viability in both cases remained above 90% (Figure 3).

**FIGURE 4:**
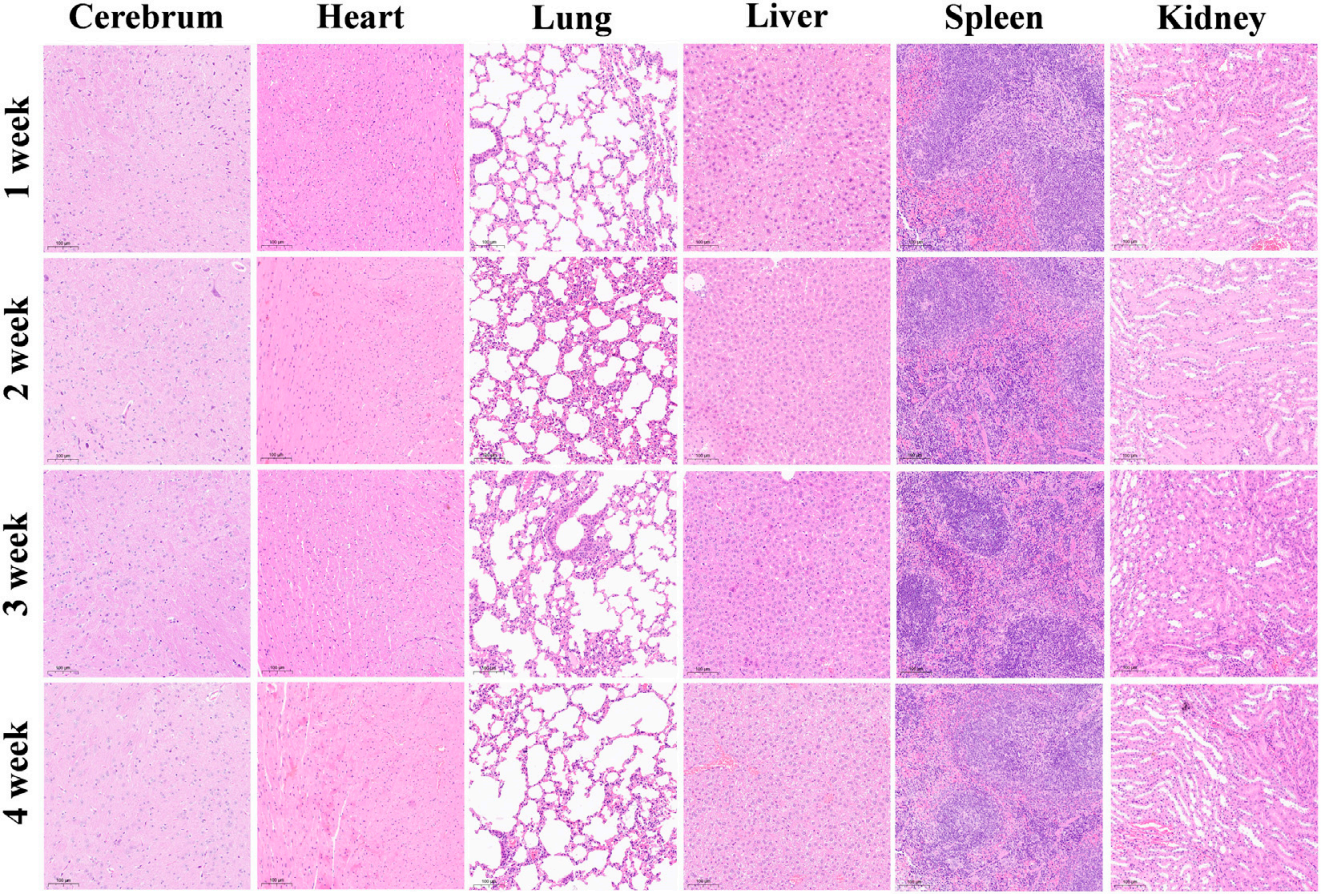
Pathological changes in the major organs of nude mice after the injection of WL-12@Fe_3_O_4_ nanoparticles, H&E staining, 10x magnification.

Hematoxylin‒eosin (HE) staining revealed no signs of necrosis, fibrosis, or other pathological changes in the major organs of the nude mice, including the brain, heart, lungs, liver, spleen, and kidneys (Figure 4).

### 3.3 Targeting properties of WL-12@Fe_3_O_4_

**FIGURE 5:**
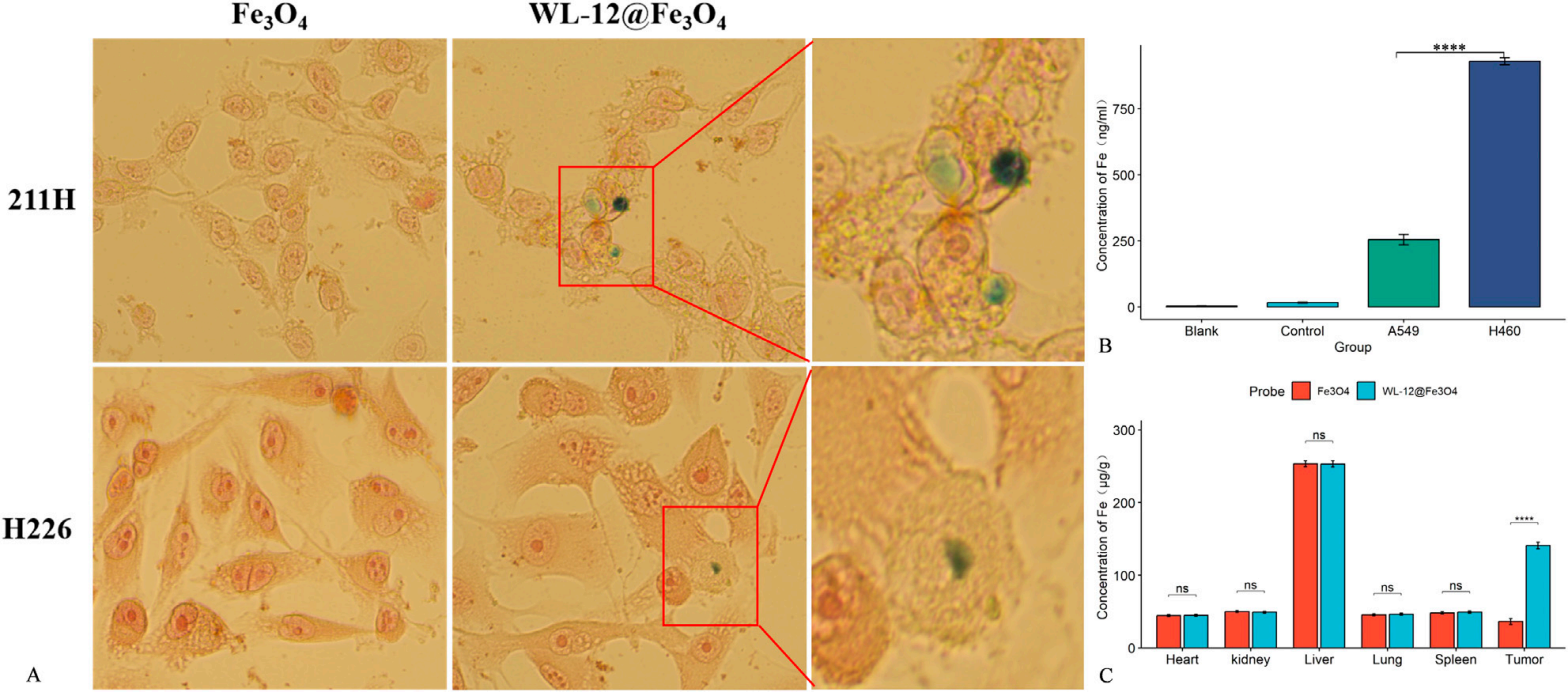
Targeting ability of WL-12@Fe_3_O_4_ nanoparticles. **(A)** Prussian blue staining and electron microscopy images of MPM cells cocultured with nanoparticles (20x magnification). **(B)** Comparison of the Fe concentration after H460 and A549 cells took up WL-12@Fe_3_O_4_ nanoparticles. **(C)** Distribution of Fe in different organs and tumor tissues after nanoparticle injection in tumor-bearing mice (ns *P* > 0.05, ****P < 0.0001).

When the Fe_3_O_4_ nanoparticles were incubated with the tumor cells, no visible blue particle deposition was observed on the tumor cells. In contrast, after incubation with WL-12@Fe_3_O_4_ nanoparticles, blue particle deposition was observed on the tumor cells, particularly in MSTO-211H cells (Figure 5A).

The concentration of iron ions in PD-L1-high H460 cells (930.22 ± 11.75 ng/mL) was significantly greater than that in PD-L1-low A549 cells (254.89 ± 17.33 ng/mL), and the difference was statistically significant (*P* < 0.05) (Figure 5B).

The iron concentration in tumor tissues injected with WL-12@Fe_3_O_4_ nanoparticles (141.02 ± 3.82 μg/g) was significantly greater than that in those injected with Fe_3_O_4_ nanoparticles (36.43 ± 3.56 μg/g, *P* < 0.05). However, no statistically significant differences in iron ion concentrations were detected between the two groups in other organs (*P* > 0.05) (Figure 5C).

### 3.4 *In vivo* MRI effects of WL-12@Fe_3_O_4_ and Fe_3_O_4_

**FIGURE 6:**
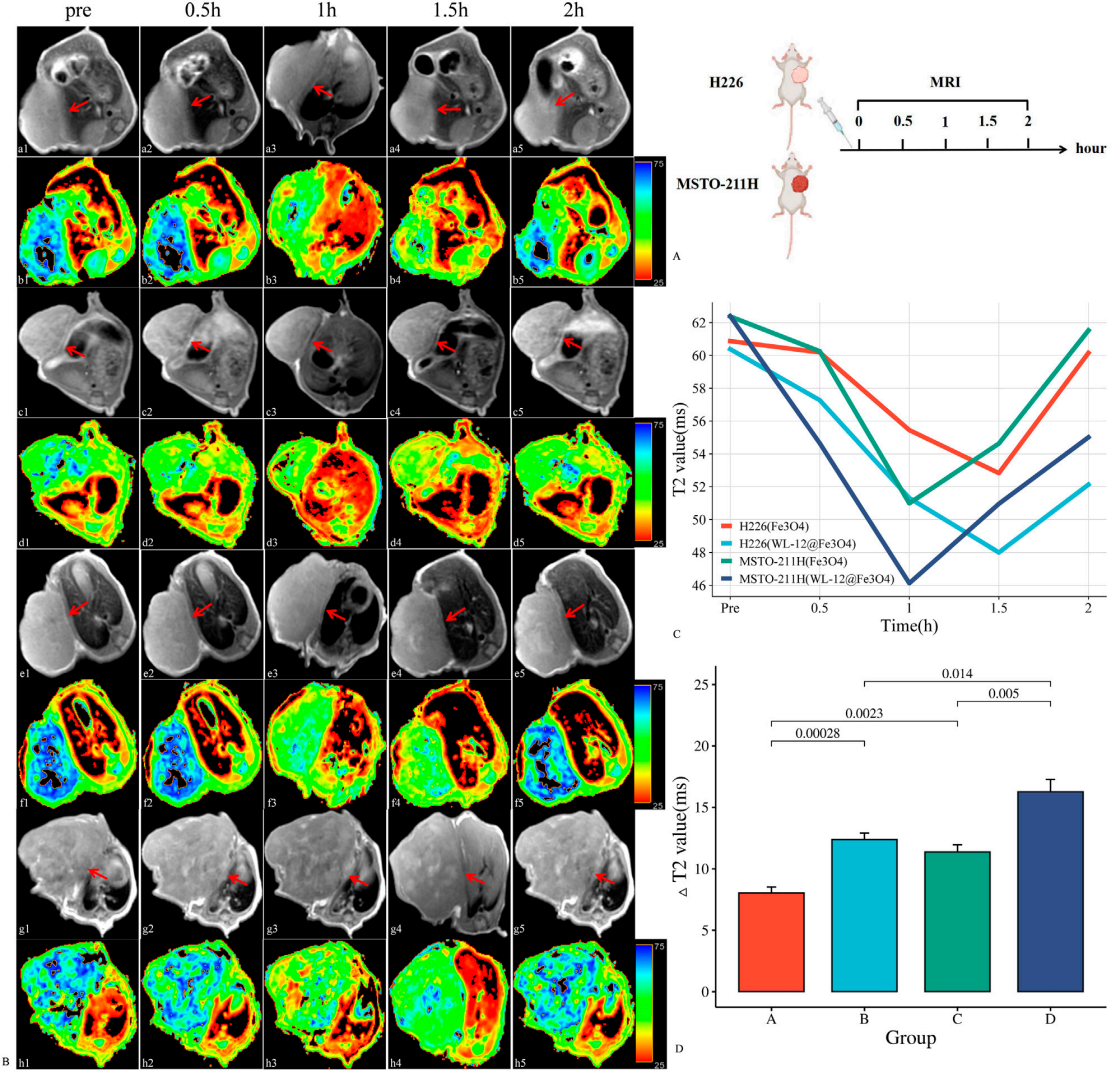
*In vivo* MR imaging analysis of WL-12@Fe_3_O_4_ and Fe_3_O_4_ nanoparticles. **(A)**. Schematic diagram of the MRI procedure in tumor-bearing mice. **(B)**. Dynamic contrast-enhanced MR images of tumor-bearing mice (red arrow: transplant tumor, including T2WI and T2 map images). (a-b: *In vivo* MRI of Fe_3_O_4_ nanoparticles in H226 tumor-bearing mice. c-d: *In vivo* MR image of WL-12@Fe_3_O_4_ nanoparticles in H226 tumor-bearing mice. e-f: *In vivo* MRI of Fe_3_O_4_ nanoparticles in MSTO-211H tumor-bearing mice. g-h: *In vivo* MR image of WL-12@Fe_3_O_4_ nanoparticles in MSTO-211H tumor-bearing mice.) **(C)**. Time‒T2 curves of H226 and MSTO-211H tumors postenhancement. **(D)**. Comparison of ΔT2 values at 1.5 h and 1 h after enhancement in H226 and MSTO-211H tumors, respectively. (A: H226 (Fe_3_O_4_), B: H226 (WL-12@Fe_3_O_4_), C: MSTO-211H (Fe_3_O_4_), D: MSTO-211H (WL-12@Fe_3_O_4_)).

After injection of the nanoparticles, the T2 values of H226 and MSTO-211H tumor tissues gradually decreased, reaching their lowest point at 1.5 h and 1 h, respectively, after which the T2 values began to increase. The T2 value changes in H226 tumors followed a “slow decrease-slow increase” pattern, whereas MSTO-211H tumors exhibited a “rapid decrease-slow increase” pattern. The ΔT2 values in the targeted group were significantly greater than those in the nontargeted group for both H226 and MSTO-211H tumors (12.38 ± 1.05 m, 16.26 ± 2.02 m vs. 8.04 ± 0.93 m, 11.38 ± 1.16 m; *P*< 0.05). Additionally, the ΔT2 values in MSTO-211H tumors were significantly greater than those in H226 tumors (*P* < 0.05) (Figure 6).

### 3.5 Targeted MRI detection of PD-L1 expression in MPM

**FIGURE 7:**
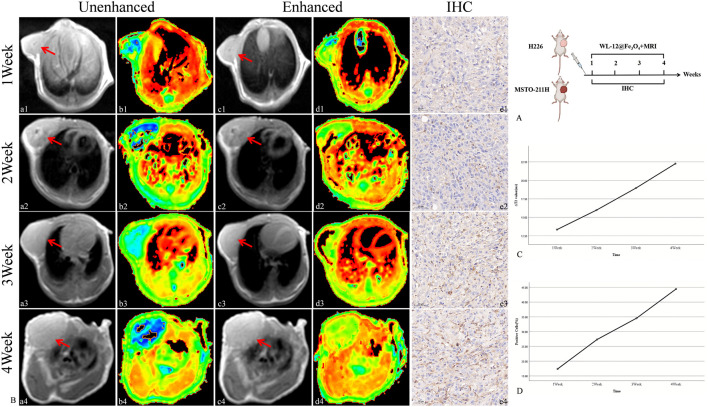
Targeted MRI detection of PD-L1 expression in H226 tumor-bearing mice. **(A)** Schematic diagram of the MRI procedure in tumor-bearing mice. **(B)** Unenhanced and enhanced MR images (red arrow: transplant tumor, including T2WI and T2 maps) and IHC images of tumor-bearing mice. (a-b: Unenhanced MR image of tumor-bearing mice. c-d: Enhanced MR image of tumor-bearing mice. e: IHC image of the tumor (20×).) **(C)** Trend of ΔT2 values in tumors. **(D)** Trend of the PD-L1-positive cell ratio in tumors.

**FIGURE 8:**
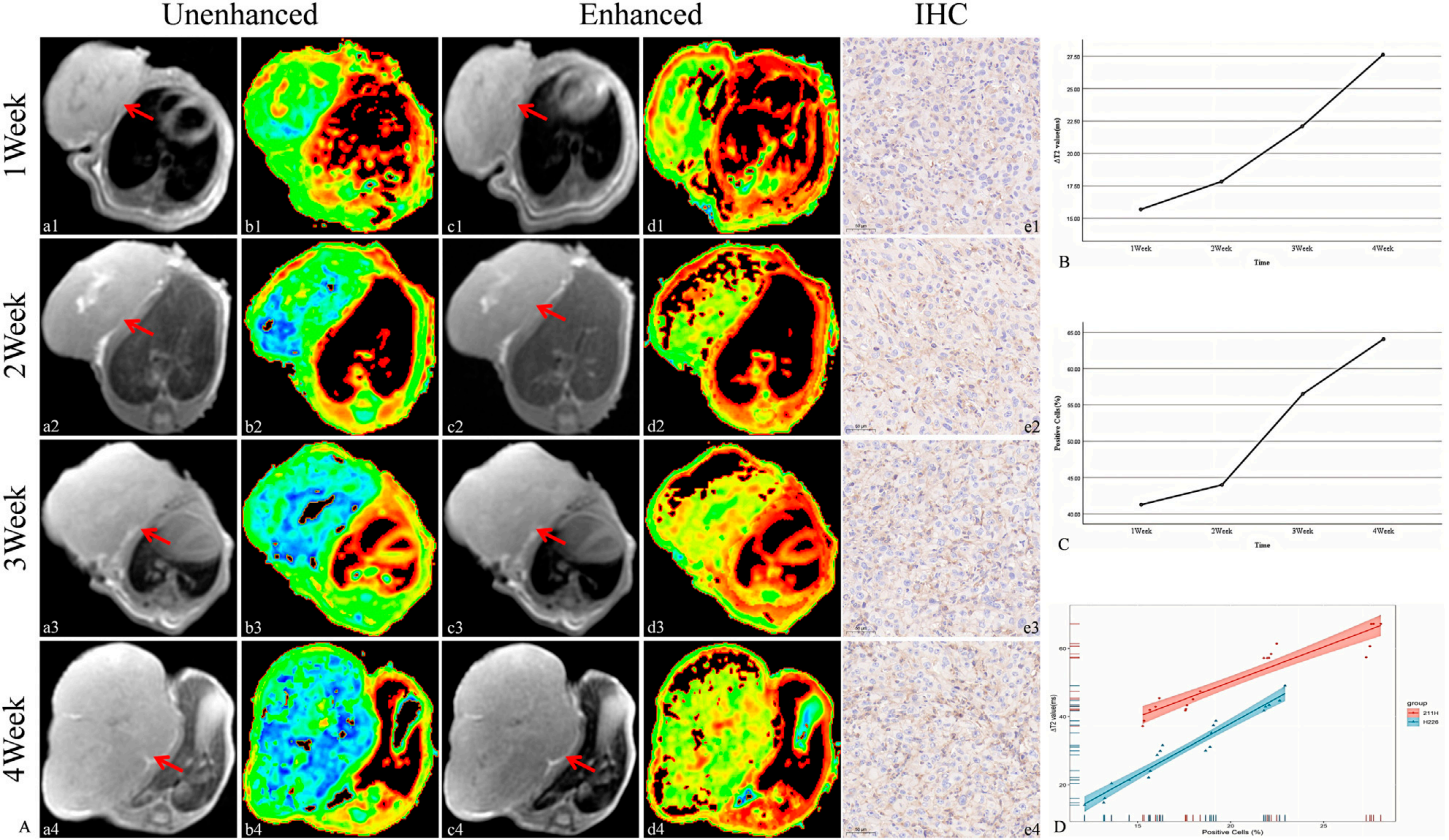
Targeted MRI detection of PD-L1 expression in MSTO-211H tumor-bearing mice and correlation analysis between ΔT2 values and the PD-L1-positive cell ratio in tumors. **(A)** Unenhanced and enhanced MR images (red arrow: transplant tumor, including T2WI and T2 maps) and IHC images of tumor-bearing mice. (a-b: Unenhanced MR image of tumor-bearing mice. c-d: Enhanced MR image of tumor-bearing mice. e: IHC image of the tumor (20×).) **(B)** Trend of ΔT2 values in tumors. **(C)** Trend of the PD-L1-positive cell ratio in tumors. **(D)** Correlation analysis between ΔT2 values and the PD-L1-positive cell ratio in tumors.

The targeted nanoparticles were injected via the tail vein, and MR scans (including T2WI and T2 mapping) were performed on H226 and MSTO-211H tumor-bearing mice prior to injection and at 1.5 h and 1 h post-injection, respectively. The results revealed that, as tumor growth progressed over time (1, 2, 3, and 4 weeks), both the ΔT2 value and PD-L1 expression of the tumor tissue increased gradually. Moreover, a strong positive correlation between ΔT2 value and PD-L1 expression was observed (R = 0.97, *P* < 0.05) (Figures 7, 8).

## 4 Discussion

In this study, superparamagnetic iron oxide nanoparticles (SPIONs), composed of Fe_3_O_4_ nanocrystals, were selected as the probe carrier. The magnetic properties of SPIONs are highly dependent on their particle size. When the particle size is reduced below 100 nm, the internal magnetic domain structure transitions from a multidomain state to a single-domain state, resulting in an increase in coercivity. When the particle size is further reduced to less than 20 nm, the SPIONs exhibit superparamagnetic behavior. If the particle size is less than 3 nm, the magnetic moment weakens due to an increase in the proportion of disordered surface spins and a decrease in magnetic anisotropy, leading to paramagnetic properties (Mai et al., 2022; Hu et al., 2023). Additionally, the surface chemistry and size of SPIONs influence their biodistribution patterns and circulation time *in vivo*. SPIONs primarily accumulate in the liver and spleen (Pham et al., 2018). Studies have shown that to avoid capture by the liver and spleen and prolong the circulation time, the nanoparticle size should be less than 100 nm. On the other hand, the nanoparticle size should be greater than 10 nm to evade renal clearance. Thus, the optimal size range for intravenous injection is 10–100 nm (Xie et al., 2018; Malhotra et al., 2020). In this study, the synthesized SPION and targeted nanoprobe sizes were 32.67 nm and 43.82 nm, respectively, falling within this safe range.

To increase the water solubility, biocompatibility, and targeting capability of SPIONs, surface modification is typically needed. In this study, EDC was used as a crosslinking agent, which primarily activates carboxyl groups on the Fe_3_O_4_ surface to produce reactive intermediates. NHS acts as an activator, promoting the formation of active esters during the synthesis process. The combination of NHS and EDC establishes an efficient coupling system, effectively conjugating SPIONs with WL-12. The XPS spectra reveal the presence of Fe 2p, O 1s, C 1s, and N 1s peaks within the nanoparticles, indicating the potential effective binding of WL-12 to Fe_3_O_4_. Furthermore, the Fe 2p peak of the nanoparticles is predominantly observed around 711.5 eV. This observation, combined with XANES data, suggests that Fe is primarily in the +3 oxidation state. The FT-IR spectra display NH stretching vibration peaks at 3,453.13 cm⁻^1^ and C=O stretching vibration peaks at 1,566.86 cm⁻^1^. The presence and positions of these peaks provide evidence for interactions between the functional groups of amino acid molecules and the Fe_3_O_4_ surface. The NH groups may participate in hydrogen bond formation, whereas the shift in the C=O peak could be attributed to interactions between carboxyl groups and iron ions. The coordination bond formation between carboxyl groups and iron ions likely results in significant C=O peak shifts. The combined results of the XPS and FT-IR spectra imply that WL-12 may successfully couple with Fe_3_O_4_ through multiple mechanisms, such as hydrogen bonding or coordination bonds. The particle size distribution curve of the WL-12@Fe_3_O_4_ nanoprobe showed a single peak, indicating a relatively uniform size distribution. Additionally, the nanoprobe exhibited a high zeta potential, suggesting good stability in solution and a low tendency to aggregate or precipitate. Furthermore, when the WL-12@Fe_3_O_4_ nanoprobes were dissolved in physiological saline or serum, no aggregation or precipitation was observed in either group, further demonstrating the excellent stability of the WL-12@Fe_3_O_4_ nanoprobe.

WL-12 is a 12-amino acid cyclic peptide that specifically targets programmed death-ligand 1 (PD-L1) with high affinity. Previous studies have shown that in murine tumor models, PET/CT imaging via 68Ga-WL12 demonstrated significantly greater uptake in tumors with high PD-L1 expression than in those with low PD-L1 expression (Zhang L. et al., 2023; Xia et al., 2023). In this study, WL-12@Fe_3_O_4_ nanoprobes were cocultured with MPM tumor cells and subjected to Prussian blue staining. The results revealed blue particle deposition on the surface of MPM cells, whereas nontargeted nanoprobes did not exhibit this phenomenon. Additionally, after the incubation of WL-12@Fe_3_O_4_ nanoprobes with H460 cells with high PD-L1 expression and A549 cells with low PD-L1 expression, the iron content in H460 cells was significantly greater than that in A549 cells. *In vivo* experiments further confirmed that the iron content in the tumor tissues of the WL-12@Fe_3_O_4_ nanoprobe group was significantly greater than that in the nontargeted group, verifying its targeting capability *in vivo*. The actively targeted nanoprobes mainly accumulated in the target organs, potentially reducing toxicity.

The toxicity of a probe is an important factor in evaluating its clinical application potential. Compared with gadolinium-based and manganese-based T1 MRI probes, SPIONs exhibit superior biosafety, as iron is an essential trace element in the human body (Weng et al., 2019). Moreover, the NHS used in this study can suppress side reactions, such as carboxyl group self-reactivity. In this study, the CCK-8 assay was used to evaluate the effects of coculturing nanoprobes with tumor cells, and increasing the probe concentration did not inhibit tumor cell growth. Additionally, following intravenous injection of the nanoprobes into mice and a 4-week observation period, no necrosis, fibrosis, or signs of damage were observed in major organs, indicating that the nanoprobes had no significant toxic effects on major organs. However, further functional assessments are needed to comprehensively evaluate changes in organ function.

To further assess the ability of WL-12@Fe_3_O_4_ nanoprobes to detect PD-L1 expression in tumors *in vivo*, MRI was used to dynamically monitor the uptake of nontargeted and targeted nanoprobes in tumor tissues. To objectively quantify signal changes in tumor tissues, T2 mapping was used to obtain the T2 relaxation time (T2 value) of tumor tissues, from which the difference in T2 values (ΔT2) before and after tumor enhancement was calculated. The variation in T2 values is directly associated with the dynamic distribution and clearance of the nanoprobe within tumor tissues. After being delivered to the tumor site via blood circulation, the nanoprobe accumulates within the tumor, reaching its peak concentration, which corresponds to the lowest observed T2 value. Over time, the probe is gradually cleared from the tumor tissue or redistributed to other tissues via the lymphatic system or bloodstream. This phenomenon indicates that the accumulation and metabolism of the nanoprobe in tumor tissues are time-dependent, with the lowest T2 value typically reflecting the maximum probe concentration within the tumor. Our findings show that the lowest T2 value in H226 and MSTO-211H tumor tissues occurs at 1.5 h and 1 h, respectively. Therefore, these time points were selected as the delay times for MR enhancement in subsequent experiments. The results showed that the ΔT2 values in MSTO-211H tumor tissues were significantly greater than those in H226 tumors, which may be due to the greater vascular density and permeability effects in MSTO-211H tumors. Moreover, the ΔT2 values in H226 and MSTO-211H tumor tissues were significantly greater in the targeted group than in the nontargeted group, and MSTO-211H tumors presented higher ΔT2 values than H226 tumors did, indicating the specificity of WL-12@Fe_3_O_4_ nanoprobes for PD-L1 detection. Previous studies have shown via MRI that lipid-coated SPIO nanoparticles conjugated with anti-PD-L1 antibodies can be used to detect PD-L1 expression in glioblastomas or temozolomide-resistant glioblastomas, demonstrating that PD-L1-SPIO can specifically target PD-L1-expressing temozolomide-resistant glioblastomas in the brain (Lee et al., 2021). Other researchers have developed various MRI-targeted nanoprobes for detecting PD-L1 expression in triple-negative breast cancer (Pan et al., 2023; Liu et al., 2023), with similar findings to those in this study. Furthermore, as tumors progress, PD-L1 expression in tumor tissues increases, and ΔT2 values also increase, indicating a strong positive correlation. These results further confirm that WL-12@Fe_3_O_4_ nanoprobes have excellent *in vivo* targeting capabilities and can dynamically monitor PD-L1 expression in tumors in real time.

In conclusion, WL-12@Fe_3_O_4_ nanoprobes demonstrated excellent stability, targeting ability and biosafety, showing significant potential in tumor visualization and dynamic monitoring of PD-L1 expression. However, this study has several limitations: (1) A subcutaneous ectopic transplantation model was used in this study. To further investigate PD-L1 changes in the MPM tumor microenvironment, an orthotopic thoracic transplantation model is needed. (2) The temporal distribution of nanoprobes *in vivo* requires further exploration.

## Data Availability

The raw data supporting the conclusions of this article will be made available by the authors, without undue reservation.
